# Diffracted X-ray blinking reveals signature crystal polymorph dynamics in 1,2,3,5-Tetrabromobenzene

**DOI:** 10.1038/s41598-025-95316-z

**Published:** 2025-03-24

**Authors:** Keegan McGehee, Koichiro Saito, Ryo Fukaya, Rie Haruki, Shunsuke Nozawa, Minghao Gao, Yuji C. Sasaki, Kazuhiro Mio, Yasuo Norikane

**Affiliations:** 1 National Institute of Advanced Industrial Science and Technology (AIST), 6-2-3 Kashiwanoha, Kashiwa, 277- 0882 Chiba Japan; 2 Research Institute for Advanced Electronics and Photonics, National Institute of Advanced Industrial Science and Technology (AIST), Tsukuba, 305-8565 Ibaraki Japan; 3 Institute of Materials Structure Science, High Energy Accelerator Research Organization, Tsukuba, 305-0801 Ibaraki Japan; 4 Graduate School of Science and Technology, University of Tsukuba, Tsukuba, 305-8571 Ibaraki Japan; 5 Graduate School of Frontier Sciences, The University of Tokyo, 5-1-5 Kashiwanoha, Kashiwa, 277-8561 Chiba Japan

**Keywords:** Chemical physics, X-ray diffraction, Crystal engineering

## Abstract

In this work we report findings on the study of 1,2,4,5-tetrabromobenzene (TBB) polymorphs by diffracted X-ray blinking (DXB). Using DXB it was found that on the ms-s time scale subtle fluctuations in the d-spacing between lattice planes occurs more rapidly in the γ-phase polymorph than in the β-phase, successfully identifying another distinct characterization between them. It was also found that the rate of molecular motion spiked near the phase transition temperature beyond the typical characteristic regions. This offers additional insight on previous reports of anisotropic lattice softening and strain accumulation near the phase transition temperature that are key to the mechanically responsive properties of TBB crystals. The present study on ms-s crystalline dynamics by DXB for mechanically responsive organic crystals also serves as a guideline for future study of more complicated mechanically responsive crystal behaviors.

**Supplementary Information:**

The online version contains supplementary material available at 10.1038/s41598-025-95316-z.

## Introduction

**Fig. 1:**
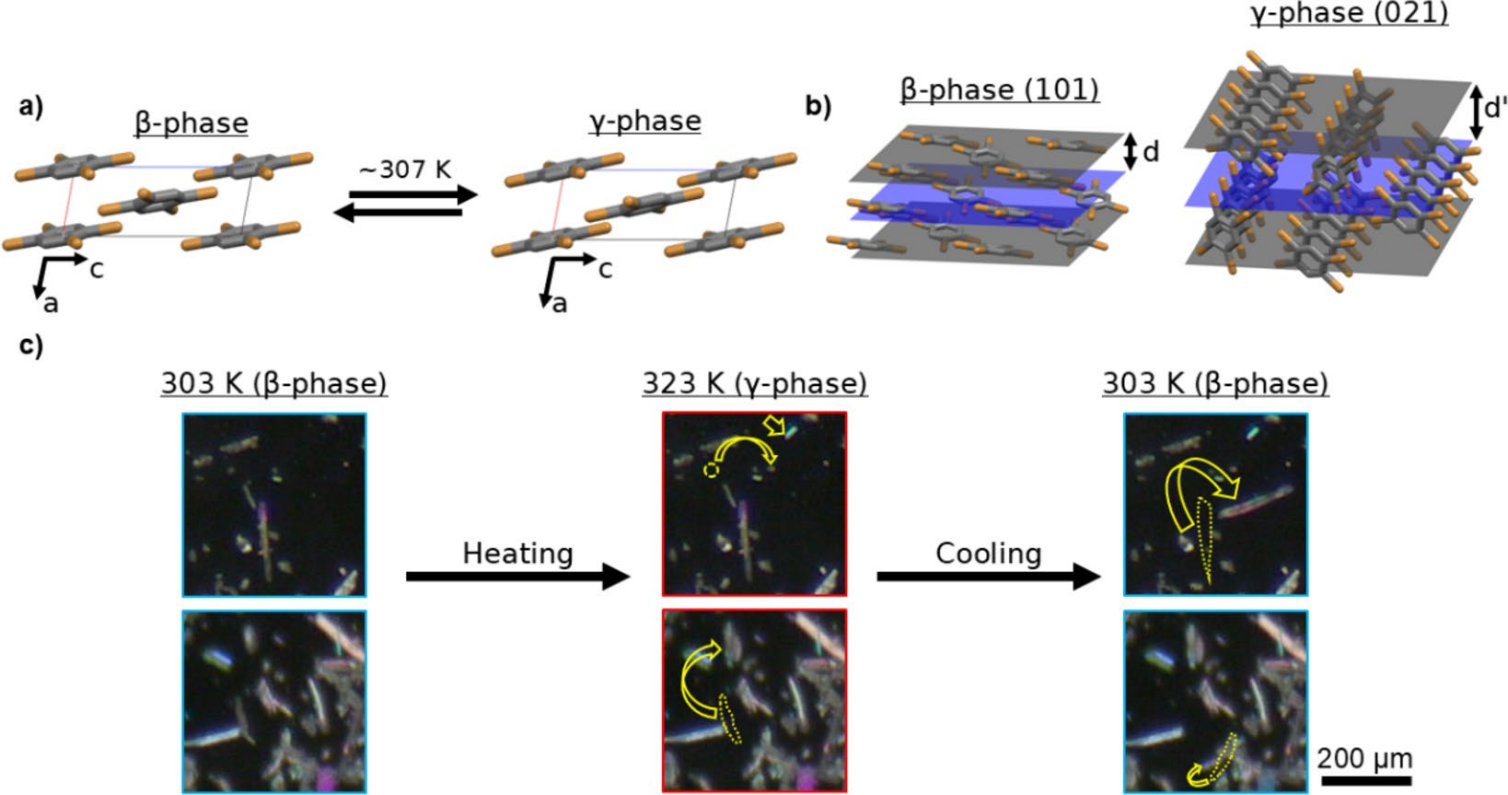
**(a)** Packed unit cells of β- and γ-phase TBB viewed normal to the (010) plane. **(b)** Example sets of lattice planes whose reflections are included in the collected XRD data. Left shows the (101) plane of β-phase, right shows the (021) plane of γ-phase. Movement of molecules along such planes correspond to analyzed intensity fluctuations. **(c)** Selected areas of unground TBB microcrystals to show thermosalient motions that may occur on reversible phase transition. Dashed yellow outlines and yellow arrows are used to indicate examples of displaced crystals. Scale bar on the far right applies to all crystal images. Taken under crossed Nicols polarization to highlight crystalline material.

Polymorphism is a property displayed by certain materials where chemically identical substances adopt distinct crystal structures. Many polymorphs are metastable under specific temperature and pressure conditions while others form under unique crystallization conditions^1–3^. The structural differences between polymorphs can give rise to a wide variation in physical properties, such as changes in solubility^4^, color^5–7^, or electronic band structure^8,9^, even though crystal packing may be minimally changed. This leads to an interest in polymorphism from a basic science and applied materials point of view. In particular, expanding tools for the study of polymorphs and transitions between them is valuable since a single point of characterization does not offer a complete view of their complexities^10^. The simple molecule 1,2,4,5-tetrabromobenzene (TBB) offers a prime example of the surprising situations found in the study of polymorphs. At lower temperatures TBB exists in the β-phase polymorph and transitions to its γ-phase polymorph at higher temperatures (Fig. 1a). This phase transition has been most precisely measured to occur at 307 K^11,12^, but has also been reported as high as 320 K depending on various experimental conditions^13–15^. The reason that crystals of this common synthesis building block^16^have gained significant attention in recent years is due to the thermosalient effect (jumping response from heat)^17^ observed on phase transition between the polymorphs (Fig. 1c). Following the initial observation of this property of TBB by Davey’s group in 2000^13^ and renewed interest sparked by Naumov’s group in 2013^14^there have been several reports on the mechanism of TBB’s phase transition^12,15,18^. Recently, specialized crystallization conditions and experimental design have even been used to create a reusable buckling microactuator with TBB crystals^19^. These studies fit into a general renewed interest in stimuli mechanoresponsive organic crystals over the past decade^20,21^ and have made TBB an important compound in this field.

Within the field of mechanically responsive crystals a growing number of examples have been found of crystals where polymorphism plays a critical role in their properties^22–26^. With many such examples involving more complex geometry and intermolecular interaction factors than the relatively simple case of TBB, the need for studying mechanically responsive crystal polymorphs with new techniques is clear. One method that is well suited to contribute to polymorph and mechanically responsive crystal studies is diffracted X-ray blinking (DXB)^27,28^. DXB is a method that was originally developed as an extension of the diffracted X-ray tracking (DXT) method, both initially for the time-resolved study of picometer scale biomolecular motions^29,30^. In more recent years DXB and DXT have also gained increased use in the study of materials^31–34^. Both methods offer valuable insight on otherwise difficult to measure rates of motion at the atomic scale, with DXB specifically being able to measure subtle fluctuations in the d-spacing between lattice planes in crystals over time. Since many of these spacings are extremely similar across TBB polymorphs (Fig. 1b) information on differences in rate of motion across the phases could be an important signature to some material properties. Here we have used DXB to study the slow (ms-s) structural dynamic properties of TBB in order to show how DXB can fit into the study of polymorphic mechanical crystal materials.

## Results

**Fig. 2:**
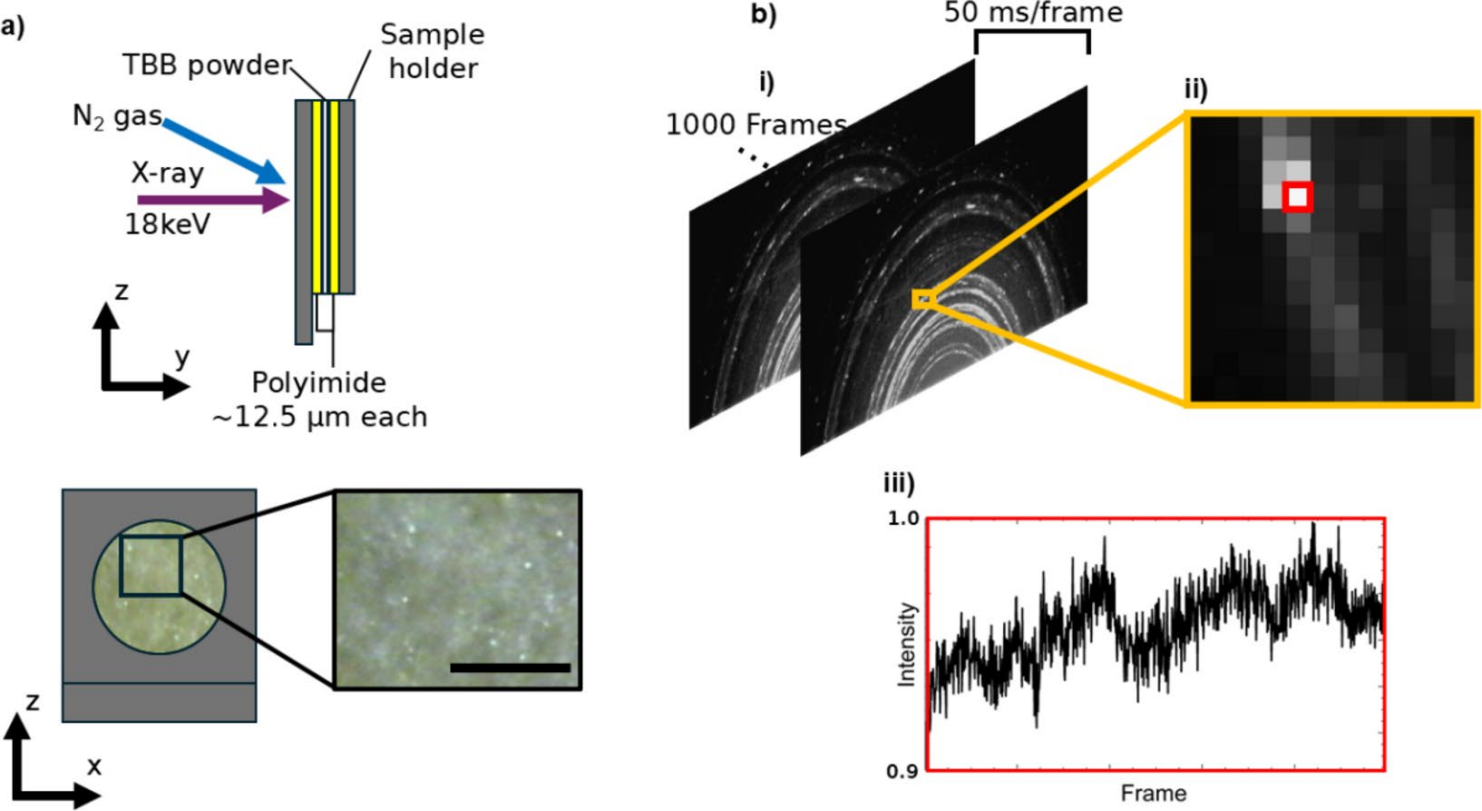
**(a)** Schematic view of the sample used in these experiments and the experiment geometry. Note cooling gas incidence is not drawn to scale for label clarity. Scale bar on the packed TBB powder image is 200 μm. **(b)** Overview of the data collection and important features. **(i)** Representative XRD images from the 2D detector. **(ii)** Zoomed in area showing the pixel structure of the images in detail. **(iii)** Relative intensity over time of the example pixel highlighted in red on **(ii)**. After background correction such signals are used for DXB analysis.

To study TBB using DXB, a powdered sample (median grain size ~ 6 μm x 13 μm) was prepared as shown in Fig. 2a. A powder was used for two main reasons. One, the phase of the sample under a given condition can be easily determined by comparison to simulated powder X-ray diffraction (XRD) patterns from reference structures. Two, collecting diffraction data from many microcrystals at once in this way improves pixel statistics for DXB analysis. An added benefit is that sufficiently small crystals generally cannot accumulate enough stress to produce significant thermosalient displacements^14^, which is beneficial for long term observation of the crystals. By packing the powder tightly any potential signal loss from thermosalient displacement should be further negated (Fig. 2a). This sample was placed under a range of temperature conditions to determine the general trend of molecular motions within TBB crystals with temperature. Ultimately to find distinct regions corresponding to each polymorph, similar to what one sees in the trend of TBB lattice parameters with temperature^13^. The tested temperature conditions will be discussed here in terms of the observed surface temperature of the TBB sample, T_s_, which we note is slightly different from the gas temperature, T_g_ (Table S1), set on the temperature controller due to heat exchange between the sample and its surroundings. Preliminary experiments showed that while no significant sample decomposition was found during one period of 50 s X-ray exposure, 2–3 periods induced noticeable yellow discoloration in the irradiated area. Thus, a fresh area of the sample was irradiated at each temperature condition by adjusting its position in the xz-plane (Fig. 2a). Diffraction data from each measurement was collected on a 2D imaging detector as shown in Fig. 2b, which was then used for polymorph phase assignment followed by DXB analysis.

**Fig. 3:**
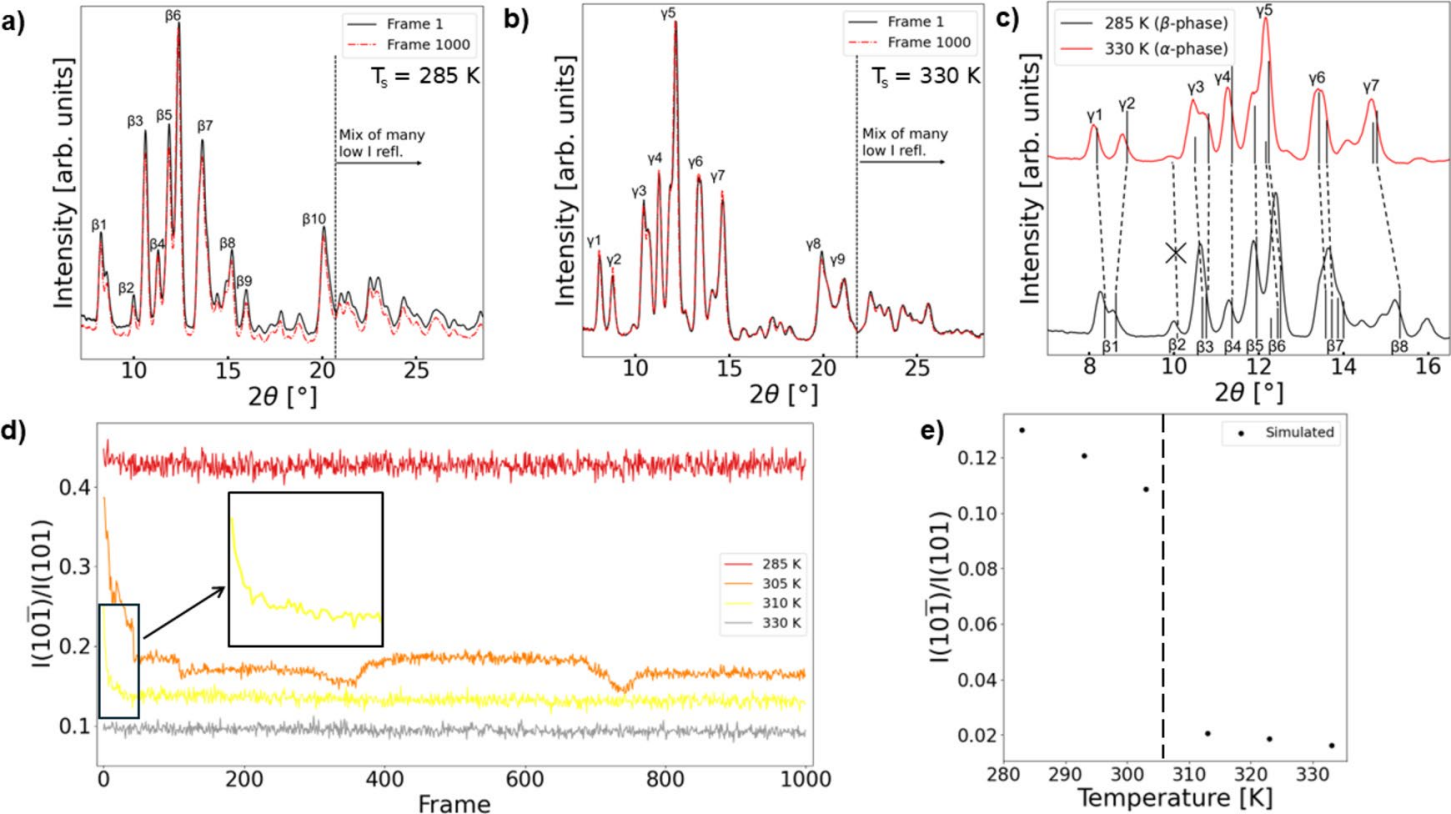
**a-b)** Diffraction patterns used to assign observed pure phase peaks for the β-phase (**a**) and γ-phase (**b**). A numeric indexing has been used to simplify references since most peaks have significant contribution from multiple reflections. Reflection details for each peak are given in Table 1. Comparing the first and last frames of each measurement shows that minimal sample damage occurred. **c)** Diffraction peak differences found most useful for differentiating the polymorphs under the current experimental conditions. Black vertical lines below the diffraction peaks are included as a visual guide for the predicted 2θ positions and relative intensities of the composite reflections for each peak. See Table 1 for details on the corresponding miller indices. Dashed lines between polymorph diffraction patterns indicate the shift of equivalent peaks. A crossed line is used to highlight peak “disappearance” (new relative intensity ≤ 1%). **d)** Plot of the ratio between intensity of the $\:10\stackrel{-}{1}$ and 101 reflections over time, which highlights the difference between unchanging samples (285 K and 330 K) and sample composition change over time (305 K and 310 K). Inset shows frames 1–50 of the 310 K measurement to aid in visualizing this rapid change. **e)** Simulated data showing the expected trend of the I($\:10\stackrel{-}{1})$/I(101) ratio with temperature. Structures with reference numbers CCDC 1578619–1578624 were used to simulate the relative intensity ratios. They correspond to crystals in the temperature range 283–333 K in 10 K increments. The vertical dashed line at 307 K indicates different polymorph regions on either side. Difference in the absolute values of the experimental and simulated ratios are likely due to inflation of I($\:10\stackrel{-}{1})$ values due to the polyimide background and imperfect sample grinding.

Following collection of the initial XRD data, the integrated diffraction patterns were examined to determine the phase composition at each temperature and assess potential sample damage. The two temperature extremes, 285 K and 330 K, were used as references for pure β-phase (Fig. 3a) and pure γ-phase (Fig. 3b), respectively. It was confirmed that diffraction patterns at these temperatures agreed well with simulated data apart from experimental peak maxima consistently showing slightly lower 2θ values (Fig. 3c, Table S2,S3). Key indicators of the phase change are shown in Fig. 3c. By comparing peak intensities at the first and last collected frames we also find that no significant sample damage occurred during measurement, which was true at all temperature conditions (Fig. S3). Within the sensitivity of these measurements, it was found that the sample at 290 K, 295 K, and 300 K also appeared to be β-phase for the entire measurement. Correspondingly, the 315 K and 320 K measurements appeared to be γ-phase. On the other hand, the experiments at 305 K (Fig. S3d) and 310 K (Fig. S3e) clearly showed phase mixtures which were not constant in time. To efficiently communicate the difference in types of sample composition over time, the ratio of intensity at the $\:10\stackrel{-}{1}$ peak (I($\:10\stackrel{-}{1})$, β2) to intensity from the 101 peak (I(101), β4 and/or γ4) at each frame for the 285 K, 305 K, 310 K, and 330 K measurements were plotted in Fig. 3d. Specifically the maximum intensity in the 2θ region of 9.49° – 9.99° was used for I($\:10\stackrel{-}{1})$, and the maximum intensity in the 11.28° – 11.30° region was used for I(101). These peaks were chosen because the $\:10\stackrel{-}{1}$ (β2) peak effectively disappears in the γ-phase while the 101 peak (β4 or γ4) intensity increases compared to the β-phase. Therefore comparing this ratio shows a high contrast between polymorphs. Additionally, since intensity contribution to these 2θ regions can be assigned to single reflections for both phases, the experimental results can be easily compared to simulated expectations (Fig. 3e).

Looking at this data it is clear that apart from noise the pure phase examples (285 K and 330 K) show no change in this ratio and show reasonable β-phase (0.4273 ± 0.0086) and γ-phase (0.0948 ± 0.0046) values. On the other hand, the 305 K and 310 K measurements transitioned from a more β-phase like high ratio to a more γ-phase like low ratio. The starting ratio for the 305 K experiment was 0.3866 and fell to a mean of 0.1720. Corresponding numbers for the 310 K experiment were 0.2508 and 0.1329, respectively. The 305 K ratio not reaching a clear steady state compared to the 310 K ratio may be a sign of a fluctuating phase mixture rather than a straightforward transition. It is reasonable to find these two temperatures as clear examples of phase transition given they are near the reported 307 K transition temperature^11^. Additionally, the sample’s T_s_was below the temperature of the incident gas. It is somewhat surprising that the sample changed upon X-ray exposure since at least two minutes of equilibration time was given to the sample at each temperature. However, it has been noted that TBB held close to the polymorph transition temperature may maintain its initial phase until given additional stimulation, for example pressure from a needle^13,14^, then transition. For organic compounds like TBB it is notably difficult to comment on the precise percent composition of arbitrary phase mixtures by powder XRD alone^3,10^, but for the purposes of the current study only the general categories of β/γ-phase or changing mixture over time are of interest. The phase assignments observed here are consistent with previous reports on TBB which confirmed neither the high X-ray flux nor the sample preparation affected the polymorph properties. This second point is particularly important as certain conditions such as nanoscale growth or significant pressure have been found to lock TBB into the γ-phase^12,35^.

**Table 1:** Primary Bragg reflections composing the indexed diffraction peaks.

Peak	hkl	Peak	hkl		Peak	hkl	Peak	hkl
β1	021, 012	β6	013, 120, $\:12\stackrel{-}{1}$		γ1	021	γ6	032, 12$\:\stackrel{-}{2}$
β2	10$\:\stackrel{-}{1}$	β7	032, 12$\:\stackrel{-}{2}$, 023, 10$\:\stackrel{-}{3}$		γ2	012	γ7	11$\:\stackrel{-}{3}$, 041
β3	110, 022	β8	041		γ3	110, 022	γ8	140, 14$\:\stackrel{-}{1}$
β4	101	β9	014		γ4	101	γ9	024, 11$\:\stackrel{-}{4}$
β5	111	β10	103, 042, 024, 11$\:\stackrel{-}{4}$		γ5	111, 120, 12$\:\stackrel{-}{1}$		

Using the collected 2D diffraction images DXB analysis was performed for each temperature. The detailed procedure has been reported previously^27,31^, but the main points and considerations for this sample are as follows. A region of interest including all pixels along the Debye-Scherrer rings for the peaks labeled in Fig. 3a-b that could be sufficiently resolved from background noise were selected for analysis. After correcting for background intensity fluctuations, the autocorrelation function (ACF) was calculated for each selected pixel using the following equation.

1 $$\:ACF\left(lag\right)=\frac{\langle\delta\:I\left(t\right)\cdot\:\:\delta\:I(t+lag)\rangle}{{I\langle\left(t\right)\rangle}^{2}}$$

Here *lag* refers to time between frames, *I(t)* refers to intensity at time *t*, and *δI(t)* refers to the difference between intensity at a given *t* and mean intensity. The 〈〉 brackets are used to indicate time averaged values. One pixel ACF functions were then fit to exponential decay functions of the following form.

2 $$\:y\left(lag\right)=A{\text{e}}^{-{\uptau\:}\cdot\:\text{l}\text{a}\text{g}}\:+\:{y}_{0}$$

**Fig. 4:**
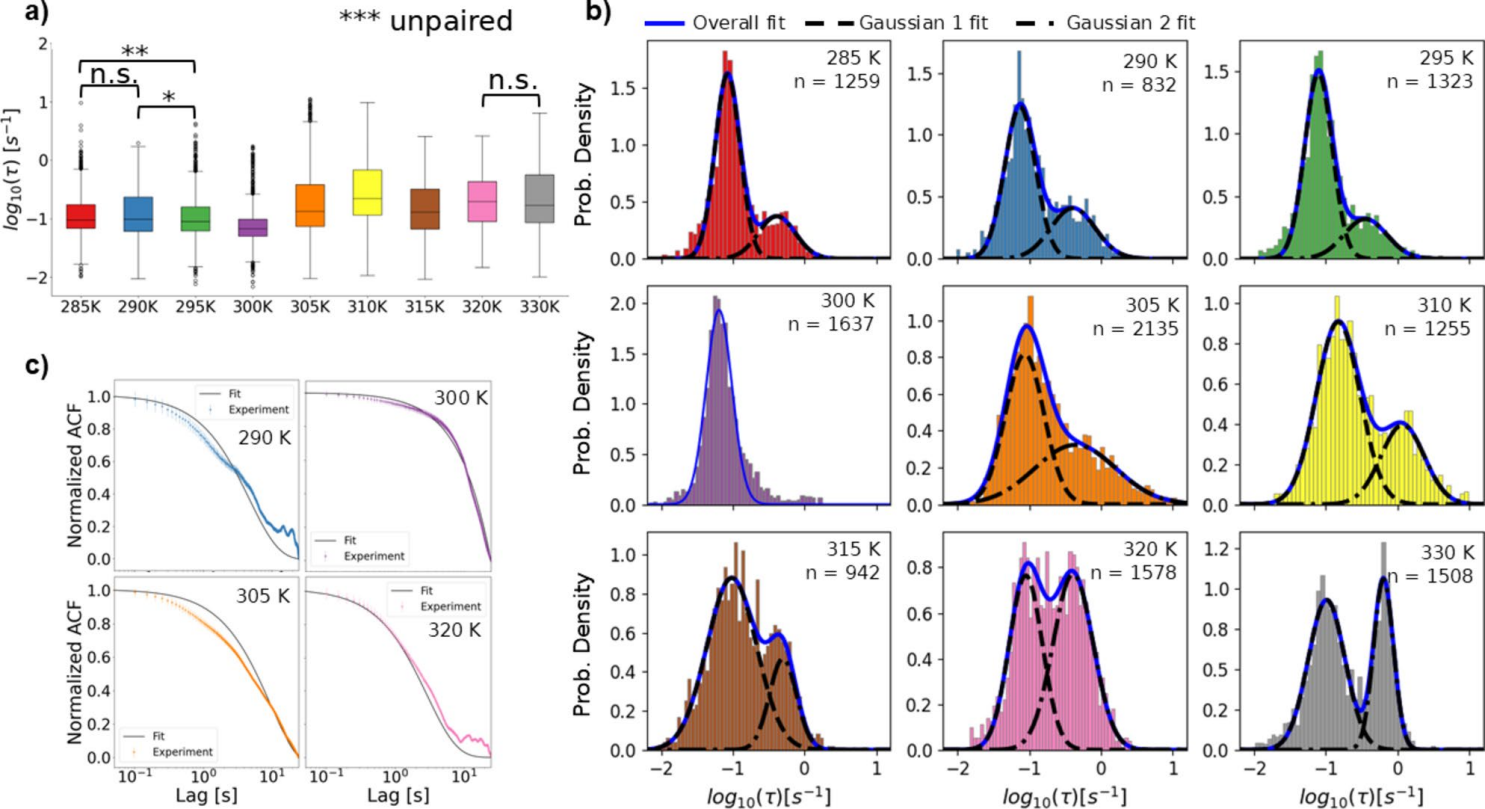
Distributions of decay constants obtained by DXB analysis at each studied temperature represented as boxplots. Boxes represent the 1st quartile (Q1), median and 3rd quartile (Q3). Whiskers are Q1 minus the interquartile range or Q3 plus the interquartile range respectively. Points beyond the whiskers may be considered outliers. Labels of n.s. (*p* > 0.05), * (0.01 < *p* ≤ 0.05), ** (0.001 < *p* ≤ 0.01), and *** (*p* ≤ 0.001) on pairs of plots correspond to results of nonparametric rank sums tests. **b)** Histograms of decay constant distributions with Gaussian function curves applied as fits. The overall fits (blue lines) are sums of two component fits, G1 (dashed black lines) and G2 (dash-dotted black lines), except for the 300 K distribution which is best described with a single Gaussian. Fit parameters are listed in Table 2. The label n indicates the number of pixels analyzed. **c)** Representative average experimental ACF data and exponential decay fits for several select temperatures

Where the constants *A* and *y*_*0*_ are taken from the experimental data and the decay constant, *τ*, is obtained by non-linear least squares fitting. Representative average experimental ACFs and fits are shown in (Fig. 4c), with other temperatures included in Fig. S6. The distribution of *τ* values obtained through this procedure allow comparison of the rate of molecular motion within the crystals at different experimental conditions. In Fig. 4a the distribution of decay constants at each temperature is shown with boxplots. While groupings of 285–300 K (β-phase), 305–310 K (mix.), and 315–330 K (γ-phase) already can be seen here, visualizing the distributions with histograms (Fig. 4b) provides additional insight. Notably, these histograms show distributions that can be reasonably well fit as a sum of two Gaussian functions (G1 at lower rates and G2 at higher rates) for most temperatures, which means using a global average value may not be the most useful way to describe them (Table 2).

The one exception being the 300 K measurement, which despite a small right-tail is much more normally distributed than the others. Since 300 K is effectively the same temperature as the experiment hutch air and this was the only condition where T_g_ = T_s_, this suggests that the higher *τ* region of the other distributions is related to heat exchange with the surrounding environment. It may be reasonable to assume that some of the more rapid *τ* values observed at 305 K and 310 K are also related to motion from phase transition.

**Table 2:** Expectation values for the decay constant, τ, extracted from Gaussian fitting of the distribution histograms under the presented analysis conditions. Gaussian fit parameters are detailed in table S4.

T_s_ [K]		50 ms lag	100 ms lag	150 ms lag	250 × 4	100 × 10
		τ [x10^−2^ s^−1^]	τ [x10^−2^ s^−1^]	τ [x10^−2^ s^−1^]	τ [x10^−1^ s^−1^]	τ [x10^−1^ s^−1^]
285	G1 G2	8.463	9.025	8.917	3.097	7.568
		41.34	35.91	31.25		
290	G1 G2	7.437	8.799	8.088	3.311	7.379
		40.58	43.93	37.88		
295	G1 G2	7.891	7.978	7.902	3.083	7.328
		35.63	27.29	40.40		
300		6.457	6.607	6.607	2.767	7.780
305	G1 G2	8.648	8.411	8.297	3.350	7.295
		43.90	38.59	38.02		
310	G1 G2	14.85	15.38	13.82	2.673	5.715
		117.5	125.9	93.04	17.95	16.90
315	G1 G2	9.699	9.207	10.12	2.992	7.129
		50.28	40.32	40.29		
320	G1 G2	8.953	9.927	11.13	3.524	7.031
		40.87	41.28	43.31		
330	G1 G2	10.25	10.50	10.10	3.698	7.145
		63.75	56.68	49.65		

**Fig. 5:**
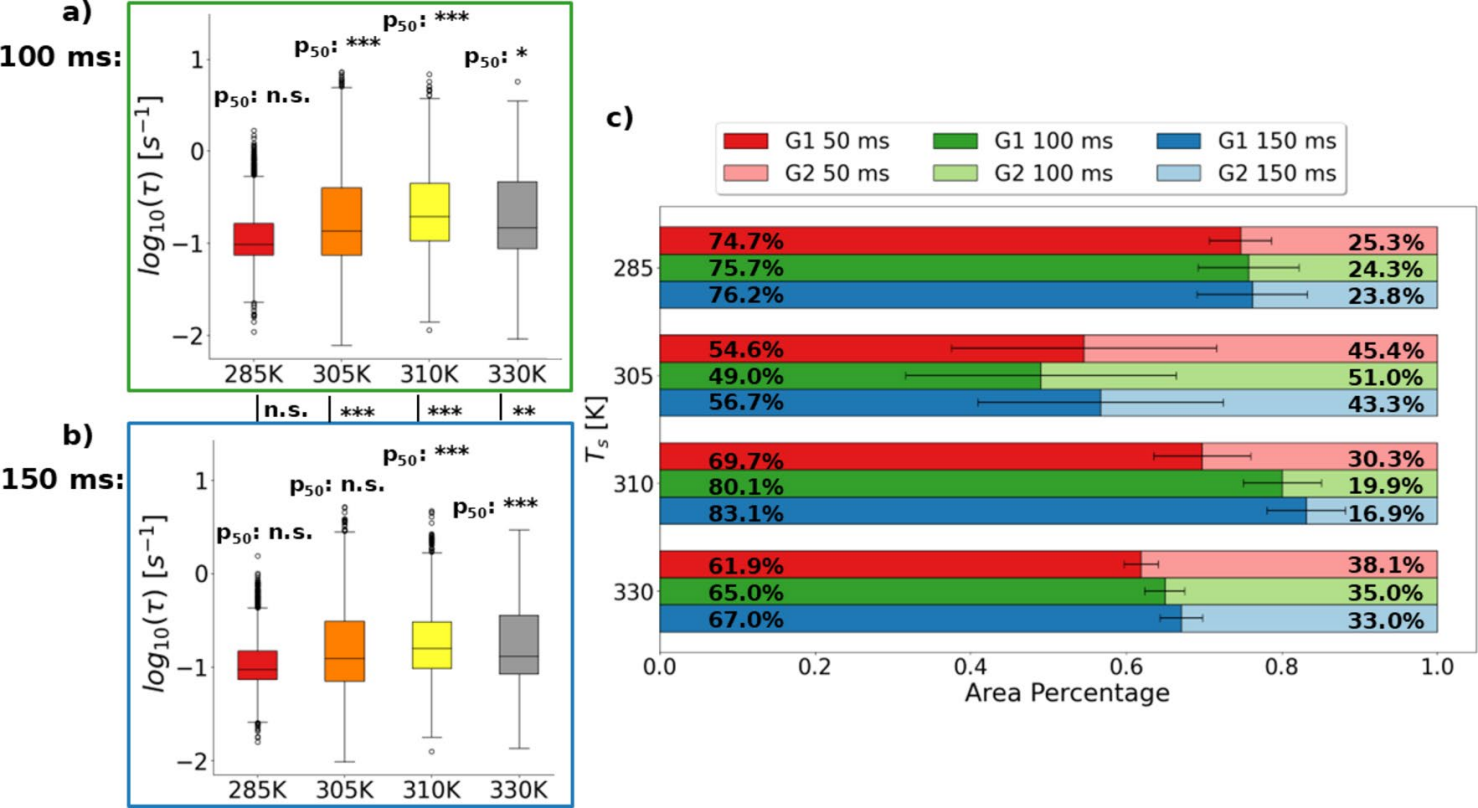
**a-b)** Boxplots showing representative τ distributions for β-phase (285 K), γ-phase (330 K), and the transitioning states (305 K and 310 K) for DXB analysis of the data binned for 100 ms (**a**) and 150 ms (**b**) effective lag. Boxes represent the 1st quartile (Q1), median and 3rd quartile (Q3). Whiskers are Q1 minus the interquartile range or Q3 plus the interquartile range respectively. Points beyond the whiskers may be considered outliers.Statistically significant difference between the different binning treatments is indicated by comparison to the 50 ms case within each set of plots (p_50_) and between the 100 ms and 150 ms cases in the space between those plots. Labels of n.s. (*p* > 0.05), * (0.01 < *p* < 0.05), ** (0.001 < *p* < 0.01), and *** (*p* < 0.001) are used as shorthand. Tests for all temperature combinations of this subset of measurements (i.e., 285 K vs. 310 K, 305 K vs. 330 K, etc.) gave *p* < 0.001 under all binning procedures. **c)** Stacked bar graph showing the area percent composition of the two Gaussian (G1 + G2) histogram fits (Fig. S11) for the representative temperatures under each binning procedure. Error bars indicate the percentage of the total area where G1 and G2 overlap.

It is worth noting that for these experiments the choice of 50 ms per frame and 1000 total frames was made primarily based on balancing time for diffraction signal accumulation per frame and overall observation time with sample damage. In other words, while our goal was to characterize the ms-s scale material properties, there is no ab initio logic to the acquisition rate, unlike in ultrafast spectroscopy for example^36^. To examine whether short term fluctuations biased any measurements and confirm the importance of total ACF time over frame rate for the observed dynamics, the data was also analyzed by binning sets of 2 and 3 frames together to create effective lag times of 100 ms and 150 ms respectively. For simplicity, the characteristic temperatures 285 K, 305 K, 310 K, and 330 K have been selected for the rest of this section. Additional results can be found in Figs. S7-16. It was found that under 100 ms and 150 ms effective lag binning no change in the general trend across polymorphs was observed (Fig. 5a-b). These *τ* distributions are also effectively fit by two Gaussians when graphed as histograms (Fig. S8,10). However, the relative area of the histograms under the two Gaussian fitting procedure was variable. These results, summarized in Fig. 5c suggest that the G2 τ region of the distribution is most susceptible to reduction when the sample changed over time. It is also clear that increased temperature leads to more change in the distribution area when the sample composition did not change over time by comparing the 285 K and 330 K cases. Since the general trend with increased binning procedures was a reduction in the G2 area, this suggests that those τ values were due to molecular motions that are near the temporal limit of detection for the current experiments. This seems to generally align with the hypothesis that the G2 region is related to heat exchange and phase transitions suggested by the lack of a G2 region at 300 K. However, the average τ values suggested by all histogram fits were minimally influenced by the frame binning procedure (Table 2). Thus p-values for statistical significance testing between the same temperature condition under different binning procedures appeared to be primarily dependent on how resistant the area percentages were to change. Ultimately the main difference between the binning procedures in this case appears to be time resolution at the transitioning temperatures, so the as collected 50 ms binning was kept for the remaining data analysis.

Having identified ranges of decay constants signature to the seconds scale dynamics of each TBB polymorph and the distinct region near the phase transition, we also examined the significance of the 50s total observation time for DXB study of these crystal polymorphs. To do this the data was analyzed in smaller sections with single frame binning, 250 frames x 4 Sect. (250 × 4) and 100 frames x 10 Sect. (100 × 10). This type of data treatment was previously used to show that the temperature dependent key dynamics of certain ice binding proteins may only be revealed by long time DXB observation^37^. Ensuring consistency in the decay constants calculated across sections also further confirms that the X-ray beam is not influencing the sample dynamics over time. The results, summarized in Fig. 5a-d suggest that for TBB polymorph dynamics the observation time dependence is more variable. Using the 250 × 4 treatment maintains the general 285 K, 305 K, 310 K, 330 K, trend at first glance (Fig. 5a), but the contrast is lower than with long time observation. The 100 × 10 treatment further flattens the trend similar to the previous ice binding protein results.

**Fig. 6:**
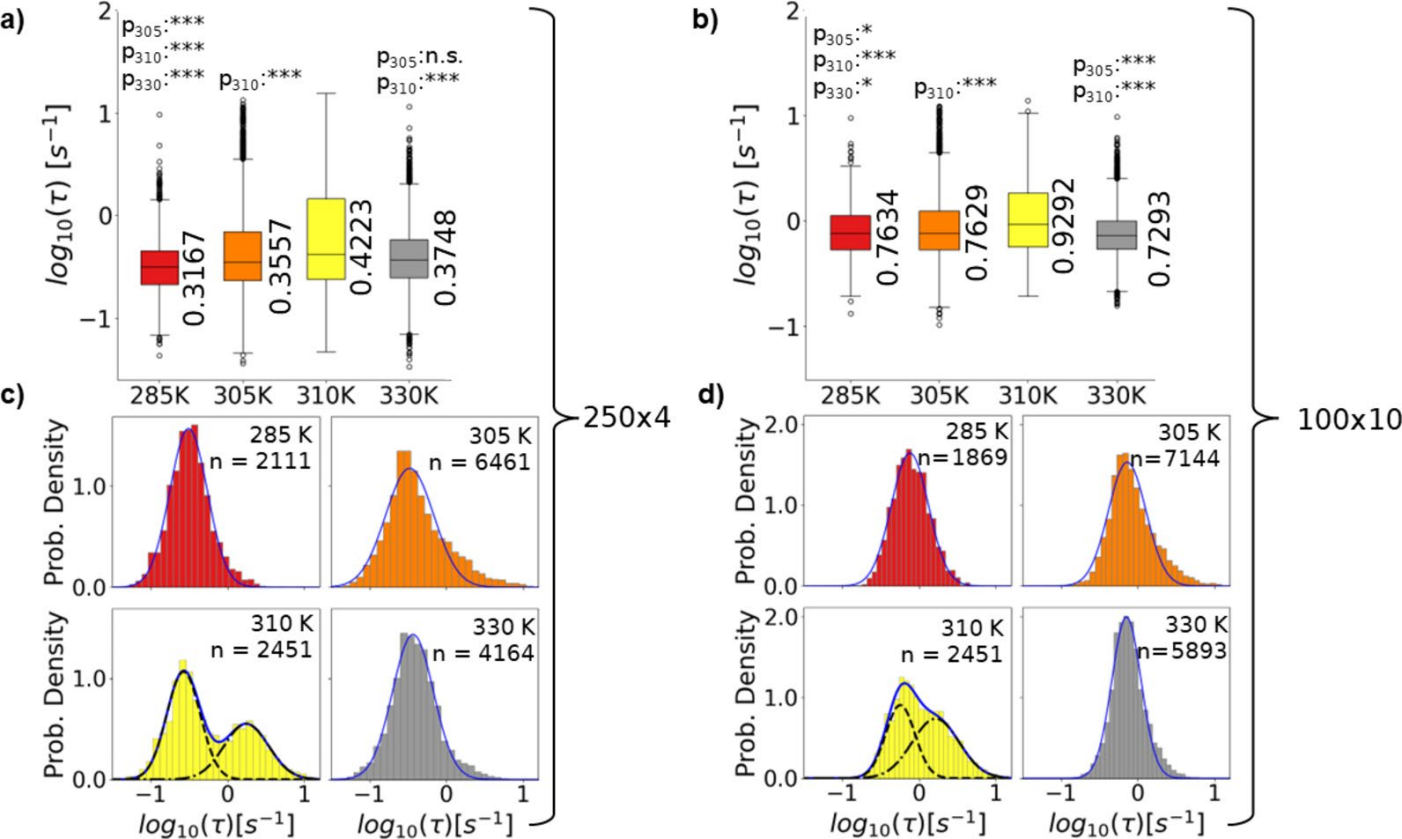
**a-b)** Boxplots for selected data analyzed in sections as 250 × 4 frames (a) or 100 × 10 frames (b). P-values between pairs of temperatures are indicated above one condition with the subscript indicating the paired condition. Boxes represent the 1st quartile (Q1), median and 3rd quartile (Q3). Whiskers are Q1 minus the interquartile range or Q3 plus the interquartile range respectively. Points beyond the whiskers may be considered outliers.

Median values of each distribution are noted to the right of the respective box. **c-d)** Histogram representations of the 250 × 4 (c) and 100 × 10 (d) distributions with one or two Gaussian functions as appropriate to produce an overall fit. Fit parameters are detailed in Table S4. The label n indicates the number of pixels analyzed.

However, the representative set was still primarily found to give statistically significantly different distributions here. Although an important note for the 100 × 10 distributions is that statistical significance tests between some β-phase and γ-phase temperatures gave p-values over 0.05 (Table S8), so, the 285 K and 330 K difference may be mainly due to temperature difference not inherent polymorph properties. Another interesting quality shown by the sectioning treatments is that the 310 K alone kept a histogram distribution of τ values best described by two Gaussians (Fig. 5c-d), with one suggesting significantly slower and the other significantly faster molecular motions than observed at the other temperatures. This is different from analyzing the full experiment time together where the entire 310 K τ distribution indicated notably faster motions. The higher decay constant region of the 310 K measurement with 250 × 4 and 100 × 10 treatment could be specifically isolated to the first 250 or 100 frames, accordingly, meaning the observed fast motions were likely phase transition. This is reasonable, but the region showing a slower rate of motion is more surprising. In the next section this point will be considered further alongside the other temperatures. Together these results suggest that while DXB can give insight on differences in TBB polymorph dynamics as well as their temperature dependence at long time observation, shorter time observation may gradually obscure some differences. The distinct behavior near the phase transition temperature for both long and short term observation stood out in particular. In the discussion section the interpretation of all observed trends will be examined in greater detail.

## Discussion

**Fig. 7:**
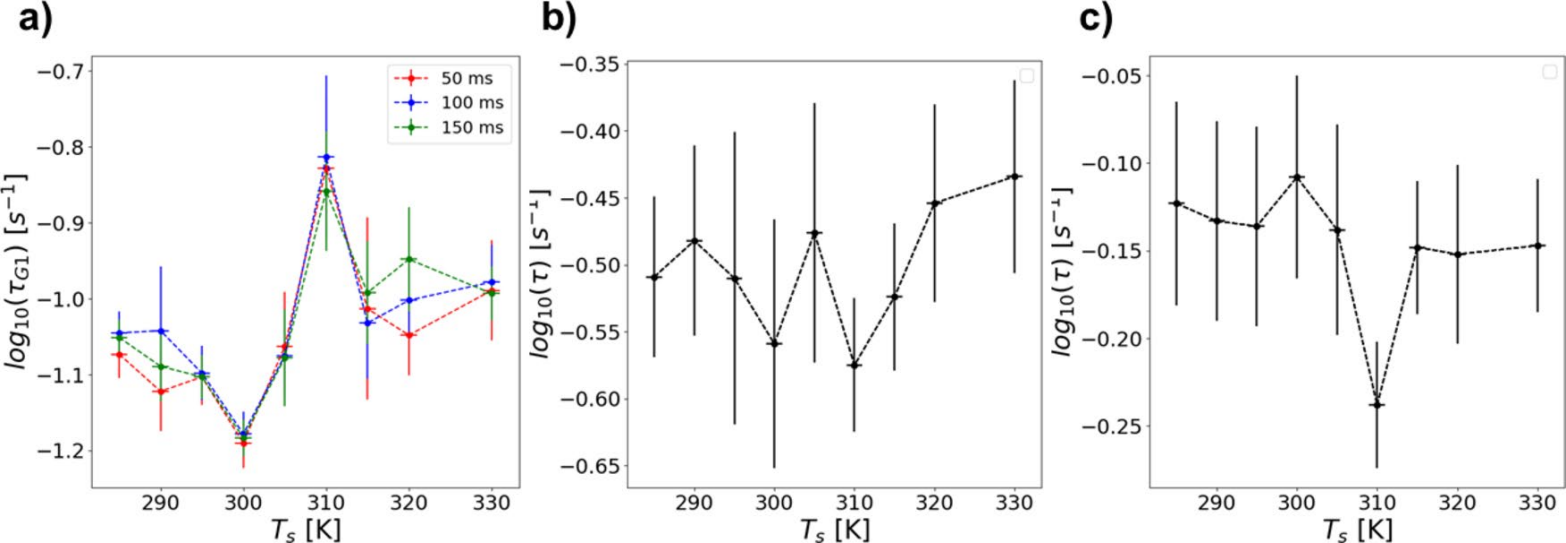
Plots of the average decay constants at each temperature given by gaussian function fitting under various data analysis treatments. Dashed lines are included as a visual guide. Error bars correspond to variance of the Gaussian fit on the y-axis and the experimentally noted temperature deviation (± 1 K) on the x-axis. **(a)** Analyzing all X-ray frames in one time series with the as collected 50 ms lag or binned to 100 ms or 150 ms effective lag. **(b)** Analyzing the data as 250 × 4 frame sections. **(c)** Analyzing the data as 100 × 10 frame sections. To allow comparison at all temperatures with each treatment, only the lower rate region (G1) is presented here for cases where two Gaussian fits were applied. Full time G2 values are presented in (Fig. 7c-d).

Considering all of the DXB results together, a picture of how this analysis complements previous TBB studies to improve understanding of the material begins to form. To visualize the difference in observed average rates of motion, the τ values suggested by Gaussian fitting procedures are shown in Fig. 7a-c. An average taken from the µ parameter of the Gaussians was chosen as a representative figure since it allows separation of the observed motion components when applicable. Additionally, the G1 parameters are highlighted here for comparison of all temperatures evenly, and because the G2 region appears generally less distinctive across conditions (Fig. S17). Looking at Fig. 6a shows the most clear trend observed in the DXB analysis of TBB polymorphs. With 50 ms binning, this long term observation of TBB shows clear characteristic τ expectation values of 7.448 ± 0.71 × 10^−2^ s^−1^ and 9.635 ± 0.53 × 10^−2^ s^−1^ for β-phase and γ-phase TBB, respectively, across the appropriate temperature ranges. The standard deviation given by looking at these values across 15 degrees of temperature variation being an order of magnitude lower than the difference in the τ values themselves is evidence that they are more sensitive to polymorph structure than temperature. In the case of temperatures showing sample change over time, a τ expectation value of 8.648 × 10^−2^ s^−1^ was found at 305 K and 14.85 × 10^−2^ s^−1^at 310 K. The 305 K value being directly between the β-phase and γ-phase values is reasonable considering the phase mixing seen at that temperature. A significantly elevated rate of motion indicated by the 310 K expectation value stands out as the most unique property shown by the DXB analysis. This increase in motion of the γ-phase near the phase transition temperature may be related to the strain accumulation that leads to the thermosalient effect^18^. These principle trends of the 50 ms binning do not change, though there are variations in the uncertainty. Particularly due to the measurements at 290 K and 320 K, (Fig. 6a) which may indicate that at these temperatures include additional sampling errors. Ultimately DXB successfully shows comparison between the molecular motions of each TBB polymorph with special behavior near the transition temperature on long time observation.

Looking at the DXB results under shorter observation time with the 250 × 4 and 100 × 10 data treatments shows that like previous studies^37^, the long observation time is import for distinguishing TBB polymorph behaviors. However, a standout expectation value is still found at 310 K for the 100 × 10 data treatment, 5.715 × 10^−1^ s^−1^ compared to 7.331 ± 0.23 × 10^−1^ s^−1^at other temperatures. Observing this slowed molecular motion rather than an increase in motion at 310 K here is likely related to the highly anisotropic nature of the TBB lattice and specifically the way that transition between its polymorphs is initiated. This has been studied in previous reports using low frequency Raman spectroscopy^12,18^and Brillouin light scattering^15^. Which showed that as TBB crystals approach phase transition, softening of the lattice leads to increased strain along the a-axis and decreased strain along the b-axis. Therefore, variation in overall DXB observation time may be more strongly correlated to the molecular motions observed in a complex crystal system than the sampling rate. One could also focus DXB analysis on specific Debye-Scherrer rings, as has often been done previously^32,34,37^, but here the strong overlap in reflections at the 2θ regions that may offer sufficient pixel statistics made the current multiplane DXB analysis more viable. Overall, this multiplane DXB observation was successful at identifying the unique molecular motion rates near the polymorph phase transition temperature under a variety of data treatments.

## Conclusions

In this study TBB crystals were studied with DXB to identify characteristic rates of molecular motion for the high and low temperature polymorphs. Unique behavior likely related to the accumulation of strain leading to the phase transition and previously reported thermosalient effect was also observed near the phase transition temperature. These distinct regions of molecular motion across polymorphs are remarkable considering the small conformational changes in TBB. The agreement found with other TBB reports shows that DXB can be a powerful tool for studying polymorphic materials with more complex behaviors in the future. Additionally, the finding that slow dynamics signatures revealed by DXB offer useful insight on mechanically responsive crystal properties is valuable for leading future work on stimuli responsive materials.

## Materials and methods

### Materials

The 1,2,4,5-tetrabromobenzene used in this study was purchased from TCI (Japan). It was recrystallized by sublimation at 80° C before use in experiments. Crystals were ground by mortar and pestle to prepare the powder used for X-ray experiments. The powder was placed between two pieces of 12.5 μm thick polyimide film and distributed in a layer on the order of 10 μm thick to prepare the final sample.

## Data collection

X-ray measurements were taken at the High Energy Accelerator Organization’s Photon Factory Advanced Ring synchrotron (Tsukuba, Japan), beamline NW14A. Details of this beamline have been published previously^38^. The beam energy was monochromatized to 18 keV. Diffraction images were taken in 50 ms intervals with 45 ms of X-ray exposure time per frame. Each experiment consisted of collecting 1000 frames. Data was collected in a transmission geometry with a sample to camera distance of 93.8 mm on a Pilatus 100 K detector (Dectris, Baden-Dättwil, Switzerland). Sample temperature was controlled with a N_2_ gas based cooling system (Cryostream, Oxford Cryosystems, Oxford, UK). The surface temperature of the sample was measured with an FLIR i3 infrared camera (Teledyne FLIR LLC, Wilsonville, OR, USA). Differences between the gas temperature (T_g_) and sample surface temperature (T_s_) are listed in Table S1.

Microscope images were recorded on a SMZ45T stereomicroscope (Nikon, Tokyo, Japan). Crystals were heated with a glass TPi-IX3-13 ThermoPlate (Tokai-Hit, Tokai, Japan) to obtain images of the thermosalient effect.

### Data analysis/software

Azimuthal integration of raw 2D detector images was done using the pyFAI package^39^. A silicon powder standard was used as a calibrant to generate the poni file. Reference TBB crystal structures, CCDC 1578619–1578624^12^, which were measured at 283–333 K in 10 K increments respectively, were used to simulate PXRD patterns from 18 keV (λ = 0.69 Å) incident X-rays in Vesta^40^. These simulated patterns were used for peak assignments and checking expected temperature dependent changes within polymorphs. Crystal structure images were created using Mercury^41^.

ImageJ^42^was used for measuring the average microcrystal size and creating masks for DXB analysis. The DXB analysis and any additional data treatment was performed using custom scripts written in Python. During DXB analysis initially selected pixels which did not maintain 90% of their original signal intensity were discarded. After ACF calculation and curve fitting, only fits with all positive coefficients were kept for further analysis. The non-linear least squares method as implemented in the SciPy^43^ package’s curve fit function was used for fitting Gaussian and exponential decay functions. Nonparametric Whitney-Man-Wilcoxon rank sums tests for statistical significance were also done using the SciPy implementation.

## Electronic supplementary material

Below is the link to the electronic supplementary material.

Supplementary Material 1

## Data Availability

Data supporting the primary conclusions of this study are presented in the main manuscript and supporting information. Additional data from this article is available from the corresponding author upon reasonable request.
