# Supplementary material for: Diffracted X-ray blinking reveals signature crystal polymorph dynamics in 1,2,3,5-Tetrabromobenzene
Source: Sci Rep. 2025 Mar 24;15:10166. doi: 10.1038/s41598-025-95316-z (PMC11933264; doi:10.1038/s41598-025-95316-z)
Supplement: Supplementary file 1 — Supplementary Material 1 [file 41598_2025_95316_MOESM1_ESM.pdf]

Diffracted X-ray Blinking Reveals Signature Crystal Polymorph Dynamics in 1,2,3,5-Tetrabromobenzene

Keegan McGehee,[1] Koichiro Saito,[2] Ryo Fukaya,[3] Rie Haruki,[3] Shunsuke Nozawa,[3] Minghao Gao,[4,1] Yuji C. Sasaki,[5,1] Kazuhiro Mio,[1] and Yasuo Norikane[* 2,4]

[1] *AIST-UTokyo Advanced Operando-Measurement Technology Open Innovation Laboratory (OPERANDO-OIL), National Institute of Advanced Industrial Science and Technology (AIST), 6-2-3 Kashiwanoha, Kashiwa 277-0882, Chiba, Japan*

[2] *Research Institute for Advanced Electronics and Photonics, National Institute of Advanced Industrial Science and Technology (AIST), Tsukuba 305-8565, Ibaraki, Japan*

[3]*Institute of Materials Structure Science, High Energy Accelerator Research Organization, Tsukuba 305-0801, Ibaraki, Japan*

[4] *Graduate School of Science and Technology, University of Tsukuba, Tsukuba 305-8571, Ibaraki, Japan*

[5]*Graduate School of Frontier Sciences, The University of Tokyo, 5-1-5 Kashiwanoha, Kashiwa 277-8561, Chiba*

*Author to whom correspondence should be addressed. Email: y-norikane@aist.go.jp

Supporting Information

Table of Contents

1. **Additional Sample and Experimental Setup Figures** 1

2. **Additional DXB Results From Binning Variation** 6

3. **Additional DXB Results from Sectioning Data Treatment** 11

4. **Analogous Plot to Figure 7 With G2 Values** 14

1. Additional Sample and Experimental Setup Figures

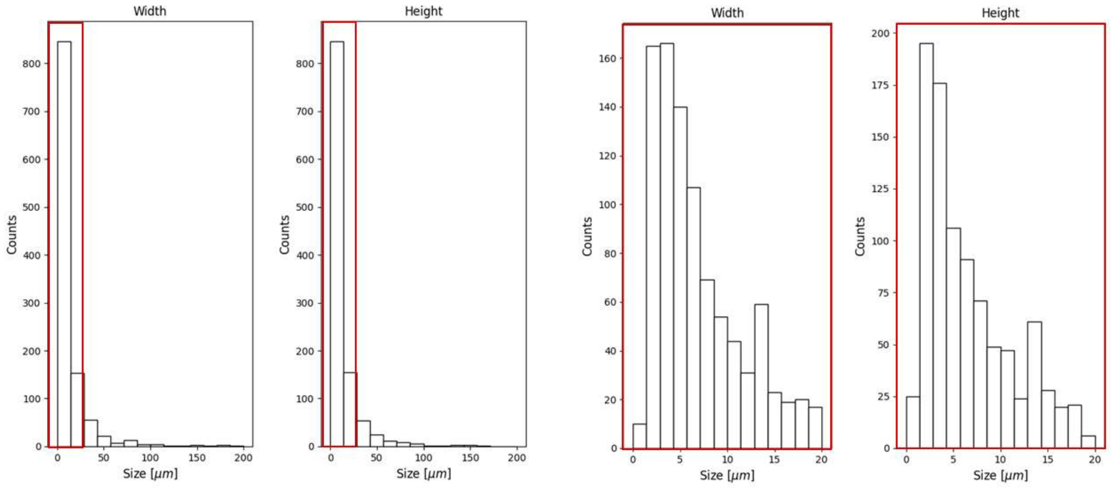

Figure S1 Microscope photos were taken of powdered TBB used in these experiments and particle analysis was used to find bounding

rectangle dimensions for estimating the powder size. The left histograms show the full size range while the right histograms highlight the distribution of most of the sample (red rectangle on left).

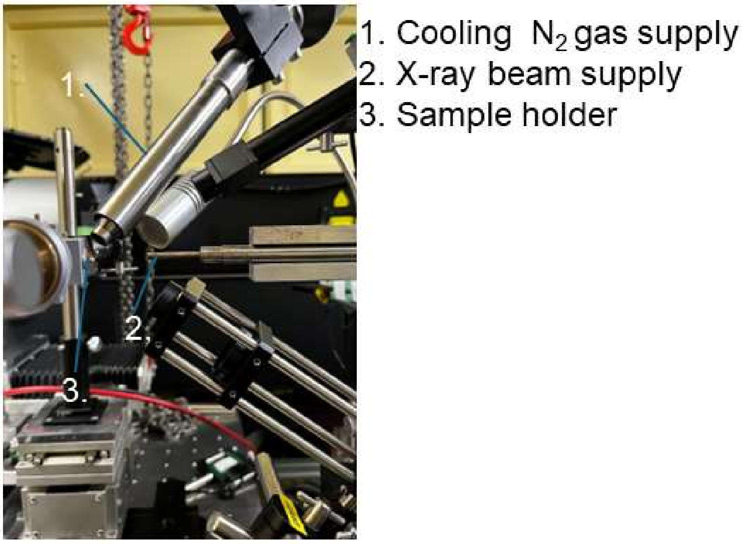

Fig. S2 Experimental setup at beamline NW14A at KEK's PF-AR synchrotron used for this study. Important features are labeled. The temperature controlling gas flow angle was adjusted as needed to optimize control at a given sample position.

Table S1 Temperature conditions tested in this study. T_g corresponds to the controlling gas temperature while T_s corresponds to the observed sample surface temperature. Expected phases are noted based on an assumption of 307 K as a transition temperature.

T_g [K]	$^*T_s$ [K]	Δ_{gs}	Expected Phase
270	285	+15	β
280	290	+10	β
290	295	+5	β
300	300	0	β
310	305	-5	β
320	310	-10	γ
330	315	-15	γ
340	320	-20	γ

*observed variation of ±1 K

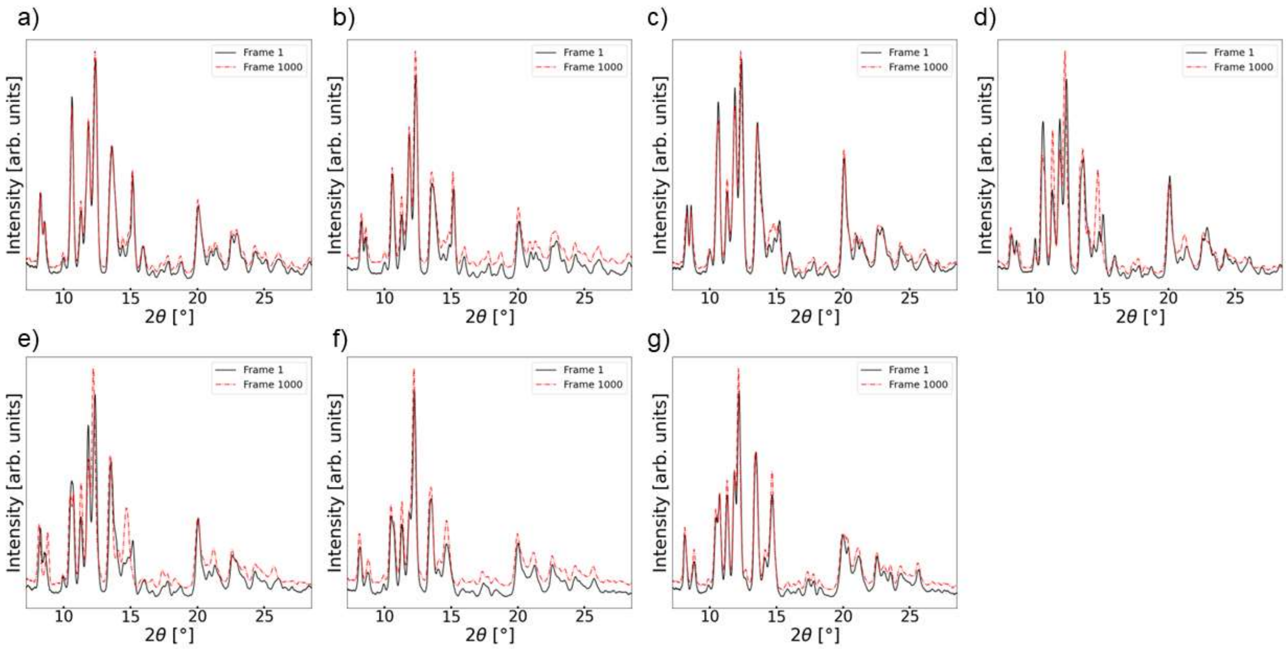

Fig. S3 First and last frame integrated diffraction patterns for measurements at; **a)** 290 k, **b)** 295 K, **c)** 300 K, **d)** 305 K, **e)** 310 K, **f)** 315 K, and **g)** 320 K.

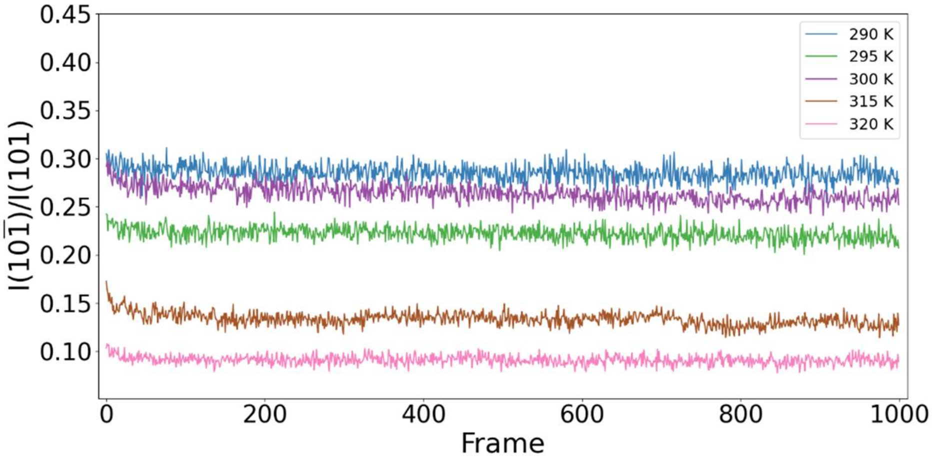

Fig. S4 Ratio between intensity from the $10\bar{1}$ and 101 reflections at additional temperatures. A ratio of approximately 0.22-0.42 corresponds with β-phase TBB while 0.09-0.15 corresponds with γ-phase TBB.

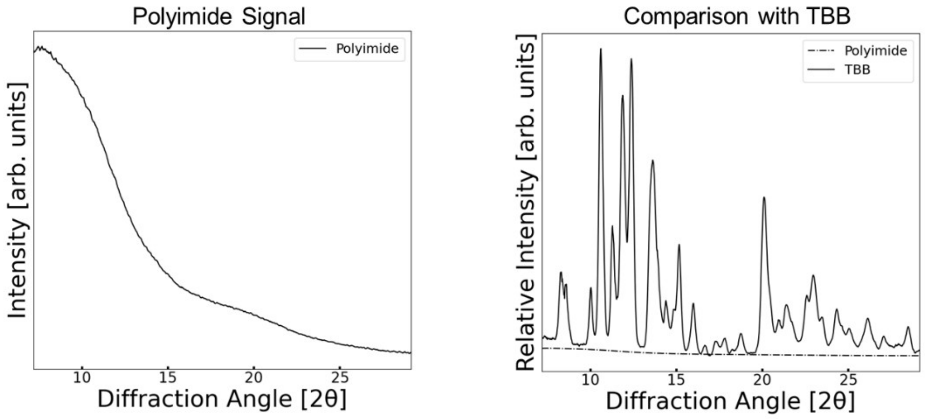

Fig. S5 Scattering signal from the polyimide film used to surround the TBB powder (left) and comparison showing the much higher relative intensity of the crystalline TBB diffraction (right).

Table S2 Details of the diffraction peak assignment for β-phase TBB at 285 K by comparison with simulated data from reference crystal structure CCDC 1578619.

Peak	hkl	2θ [°]	Peak	hkl	2θ [°]
β1		Observed 8.0-8.8	β7		Observed 13.2-14.1
	021	Calculated 8.4		032	Calculated 13.6
	012	8.6		$12\bar{2}$	13.7
β2		Observed 9.8-10.2		023	13.9
	$10\bar{1}$	Calculated 10.1		$10\bar{3}$	14.0
β3		Observed 10.4-10.9	β8		Observed 15.0-15.5
	110	Calculated 10.7		041	15.3
	022	10.8	β9		Observed 15.7-16.3
β4		Observed 11.1-11.5		014	16.1
	101	Calculated 11.4	β10		Observed 17.6-18.0
β5		Observed 11.7-12.1		$13\bar{3}$	Calculated 17.9
	111	11.9		$14\bar{1}$	18.0
β6		Observed 12.1-12.6	β11		Observed 18.5-19.2
	013	Calculated 12.3		$14\bar{2}$	Calculated 18.8
	120	12.4		051	19.0
	$12\bar{1}$	12.5	β12		Observed 19.8-20.5
				133	Calculated 20.1
				$20\bar{2}$	20.2
				142	20.3

Table S3 Details of the diffraction peak assignment for γ-phase TBB at 330 K by comparison with simulated data from reference crystal structure CCDC 1578624.

Peak	hkl	2θ [°]	Peak	hkl	2θ [°]
γ1		Observed 7.8-8.4	γ7		Observed 14.4-14.9
	021	Calculated 8.2		10$\bar{3}$	Calculated 14.7
				041	14.8
γ2		Observed 8.6-9.0	γ8		Observed 17.0-17.5
	012	Calculated 8.9		140	Calculated 17.3
γ3		Observed 10.2-10.8		14$\bar{1}$	17.4
	110	Calculated 10.5	γ9		Observed 17.5-18.0
	022	10.8		024	17.8
γ4		Observed 11.0-11.5		11$\bar{4}$	17.9
	101	Calculated 11.4	γ10		Observed 18.0-18.5
γ5		Observed 11.6-12.5		051	Calculated 18.2
	111	11.9	γ11		Observed 19.5-20.5
	120	12.1(6)		133	Calculated 20.2
	12$\bar{1}$	12.2(4)		150	20.3
γ6		Observed 13.1-13.7	γ12		Observed 20.7-21.5
	032	Calculated 13.4		151	Calculated 21.1
	12$\bar{2}$	13.6		15$\bar{2}$	21.3

2. Additional DXB Results From Binning Variation

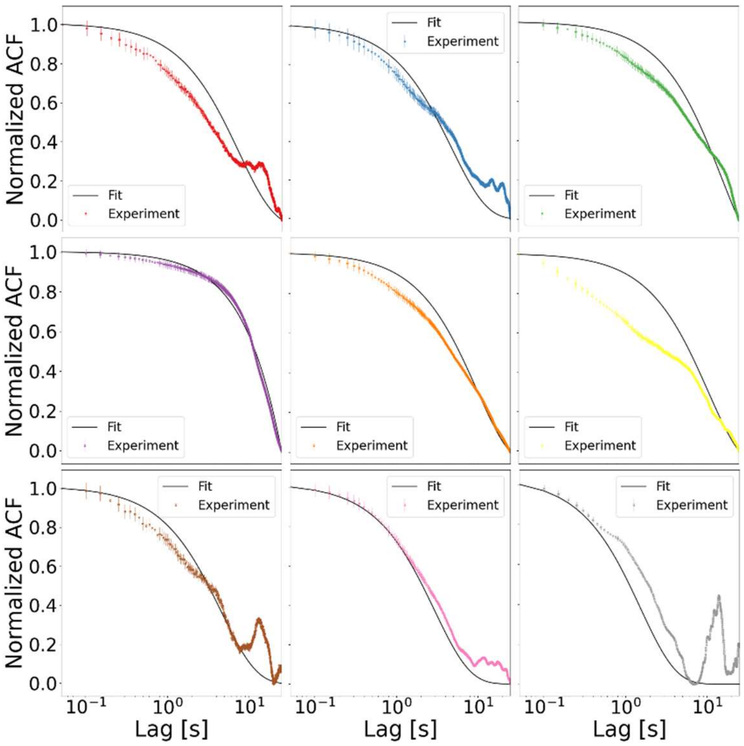

Fig. S6 Average full time experimental ACF curves and fits for all temperatures with single frame binning. Red: 285 K, blue: 290 K, green: 295 K, purple: 300 K, orange: 305 K, yellow: 310 K, brown: 315 K, pink: 320 K, gray: 330K.

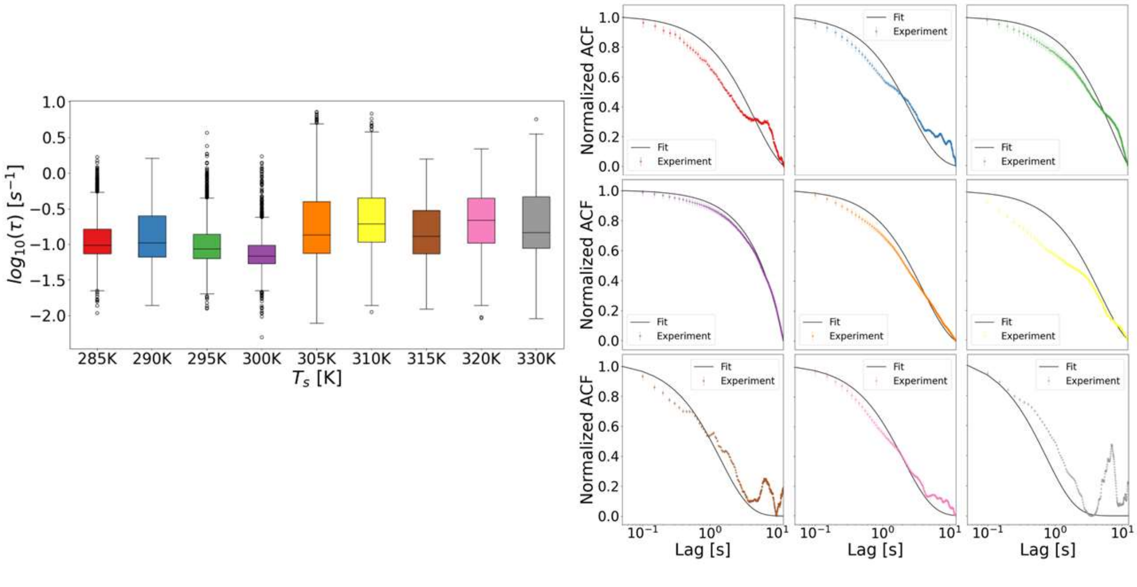

Fig. S7 Boxplots of τ values obtained by DXB analysis and average ACF curves for data analyzed with 2 frame (100 ms) binning. ACF colors match boxplots: red: 285 K, blue: 290 K, green: 295 K, purple: 300 K, orange: 305 K, yellow: 310 K, brown: 315 K, pink: 320 K, gray: 330K.

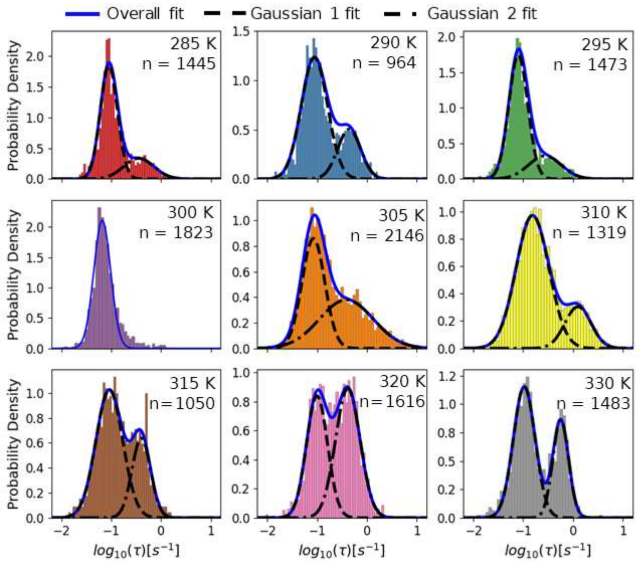

Fig. S8 Histograms of τ values obtained by DXB for data analyzed with 2 frame (100 ms) binning.

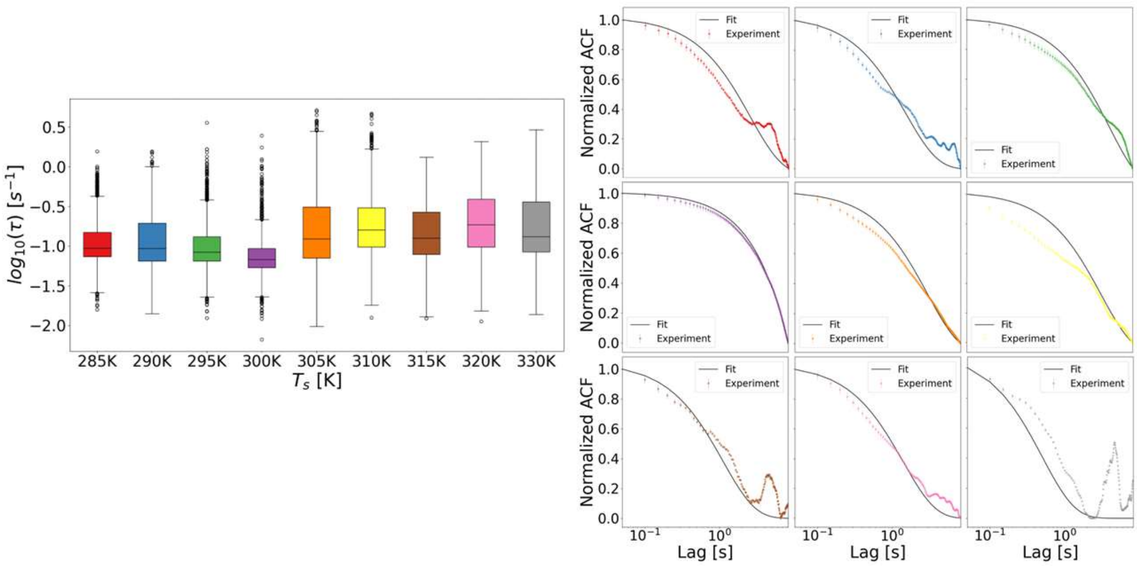

Fig. S9 Boxplots of τ values obtained by DXB analysis and average ACF curves for data analyzed with 3 frame (150 ms) binning. ACF colors match boxplots: red: 285 K, blue: 290 K, green: 295 K, purple: 300 K, orange: 305 K, yellow: 310 K, brown: 315 K, pink: 320 K, gray: 330K.

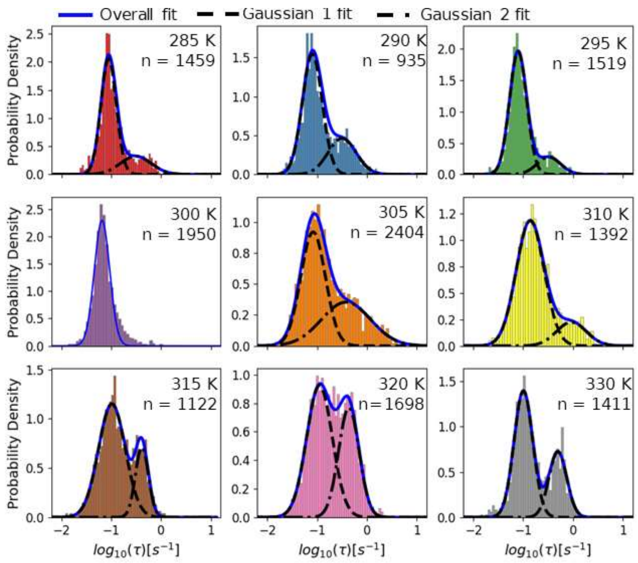

Fig. S10 Histograms of τ values obtained by DXB for data analyzed with 3 frame (150 ms) binning.

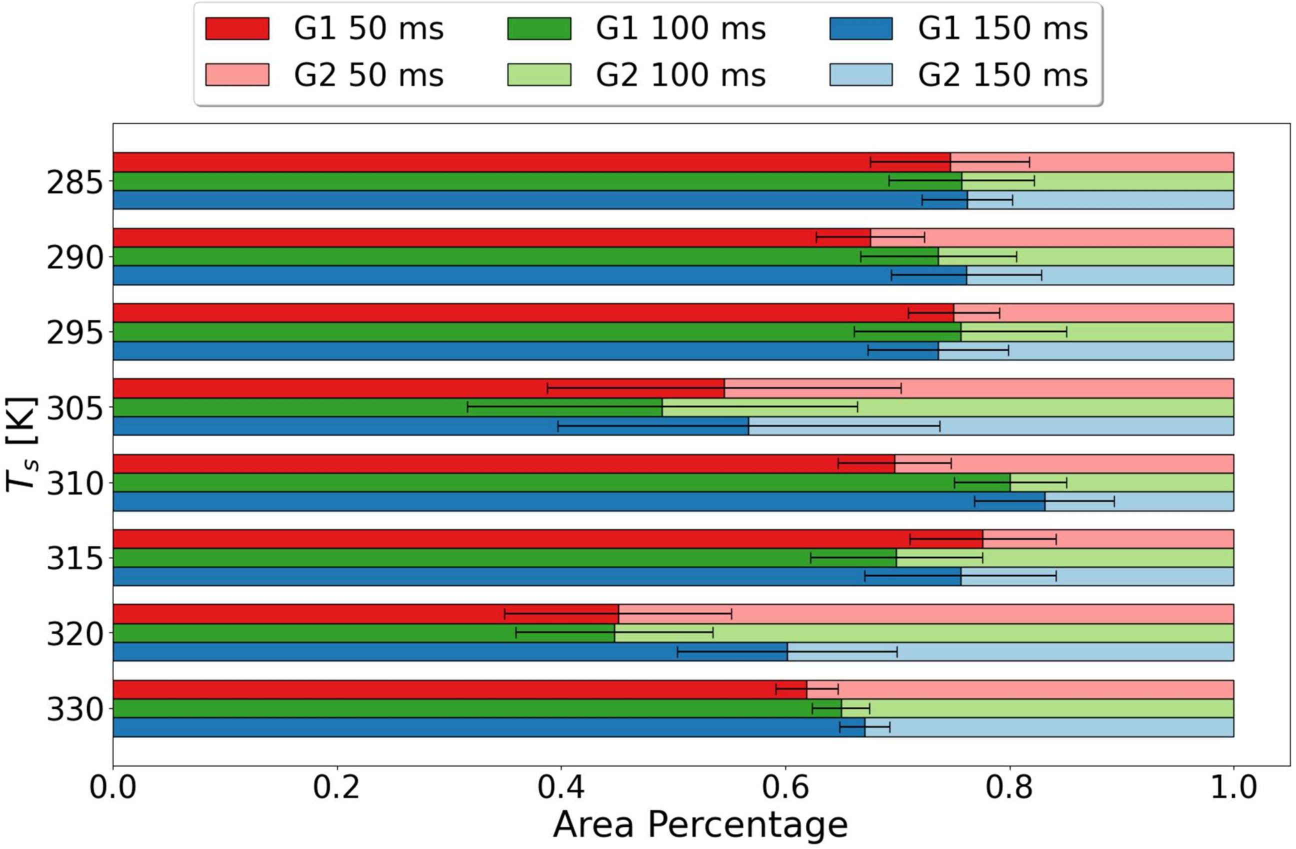

Fig. S11 Area percentages of the τ distribution histogram Gaussian fits under different binning procedures.

Table S4 Gaussian fit parameters of histograms for each temperature under various analysis conditions. Here μ indicates the average value while σ indicates standard deviation.

T_s [K]		50 ms lag		100 ms lag		150 ms lag		250x4		100x10	
		μ	σ	μ	σ	μ	σ	μ	σ	μ	σ
285	G1	-1.07	0.175	-1.04	0.171	-1.05	0.324	-0.509	0.245	-0.121	0.243
	G2	-0.384	0.257	-0.339	0.221	-0.505	0.309				
290	G1	-1.13	0.216	-1.06	0.264	-1.10	0.199	-0.480	0.266	-0.132	0.239
	G2	-0.392	0.311	-0.355	0.223	-0.500	0.282				
295	G1	-1.10	0.192	-1.10	0.190	-1.10	0.173	-0.511	0.330	-0.135	0.238
	G2	-0.448	0.298	-0.399	0.268	-0.486	0.236				
300		-1.19	0.181	-1.18	0.170	-1.18	0.155	-0.558	0.307	-0.109	0.242
305	G1	-1.06	0.268	-1.08	0.229	-1.08	0.247	-0.475	0.313	-0.137	0.245
	G2	-0.358	0.564	-0.414	0.539	-0.420	0.490				
310	G1	-0.828	0.304	-0.813	0.329	-0.859	0.247	-0.573	0.222	-0.243	0.189
	G2	0.0699	0.304	0.100	0.262	-0.0313	0.490	0.254	0.282	0.228	0.298
315	G1	-1.01	0.346	-1.04	0.269	-0.984	0.268	-0.524	0.235	-0.147	0.196
	G2	-0.299	0.188	-0.395	0.186	-0.382	0.130				
320	G1	-1.05	0.231	-1.00	0.211	-0.953	0.259	-0.453	0.271	-0.153	0.228
	G2	-0.389	0.279	-0.384	0.246	-0.363	0.206				
330	G1	-0.989	0.256	-0.979	0.218	-0.996	0.188	-0.432	0.267	-0.146	0.191
	G2	-0.195	0.139	-0.247	0.158	-0.304	0.178				

Table S5 Frame binning procedures compared with nonparametric Wilcoxon-Mann-Whitney rank sums tests for the null hypothesis that distributions at different temperatures show statistically significant differences. The shorthand n.s ($p > 0.05$), * ($0.01 < p < 0.05$), ** ($0.001 < p < 0.01$), and *** ($p < 0.001$) is used for clarity.

T_s [K]	50 ms/100 ms	50 ms/150 ms	100 ms/150 ms
285	n.s.	n.s.	n.s
290	***	***	n.s.
295	n.s.	n.s	n.s.
300	n.s	n.s.	n.s.
305	***	n.s.	***
310	***	***	***
315	n.s.	n.s.	n.s.
320	***	*	n.s.
330	**	*	***

Table S6 Full ACF time Results of nonparametric Wilcoxon-Mann-Whitney rank sums tests for the null hypothesis that distributions at different temperatures show statistically significant differences. The shorthand n.s ($p > 0.05$), * ($0.01 < p < 0.05$), ** ($0.001 < p < 0.01$), and *** ($p < 0.001$) is used for clarity.

T [K]	285	290	295	300	305	310	315	320	330
**285**	1.0	-	-	-	-	-	-	-	-
**290**	n.s.	1.0	-	-	-	-	-	-	-
**295**	**	*	1.0	-	-	-	-	-	-
**300**	***	***	***	1.0	-	-	-	-	-
**305**	***	***	***	***	1.0	-	-	-	-
**310**	***	***	***	***	***	1.0	-	-	-
**315**	***	***	***	***	***	***	1.0	-	-
**320**	***	***	***	***	***	***	***	1.0	-
**330**	***	***	***	***	***	***	***	n.s.	1.0

3. Additional DXB Results from Sectioning Data Treatment

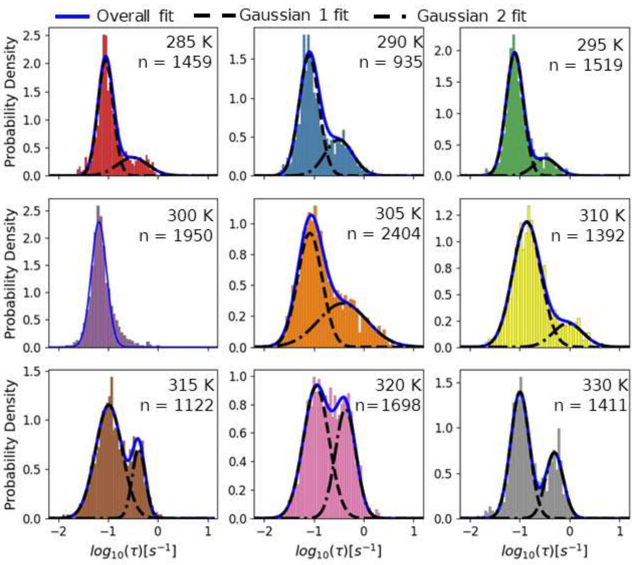

Fig. S12 Histograms of τ values obtained by DXB for data analyzed with 3 frame (150 ms) binning.

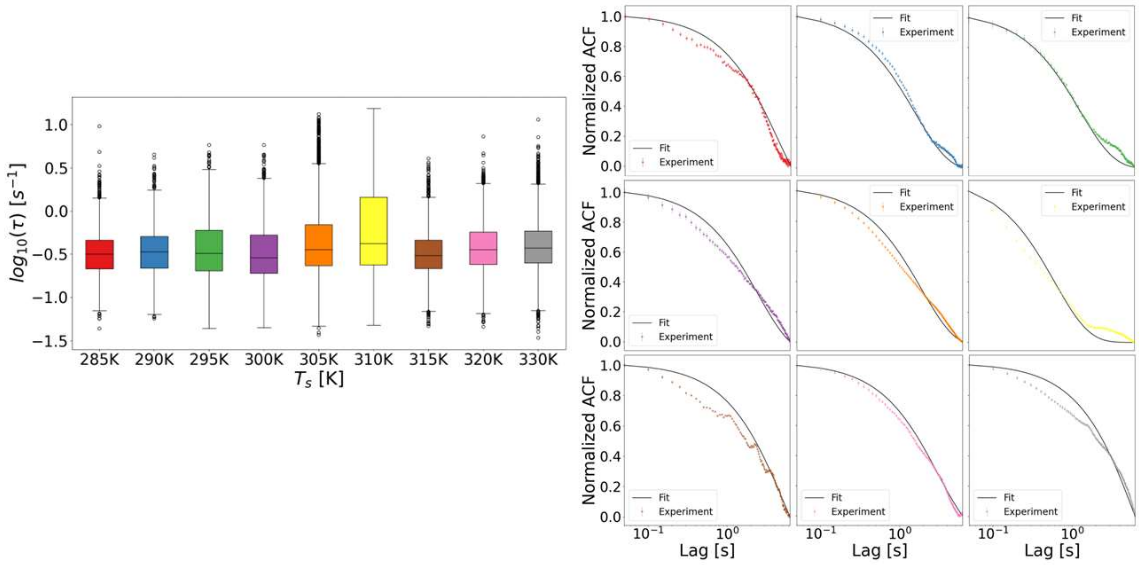

Fig S13. Boxplots for τ values and average ACF curves of DXB analysis by 250x4 treatment. ACF colors match boxplots: red: 285 K, blue: 290 K, green: 295 K, purple: 300 K, orange: 305 K, yellow: 310 K, brown: 315 K, pink: 320 K, gray: 330K.

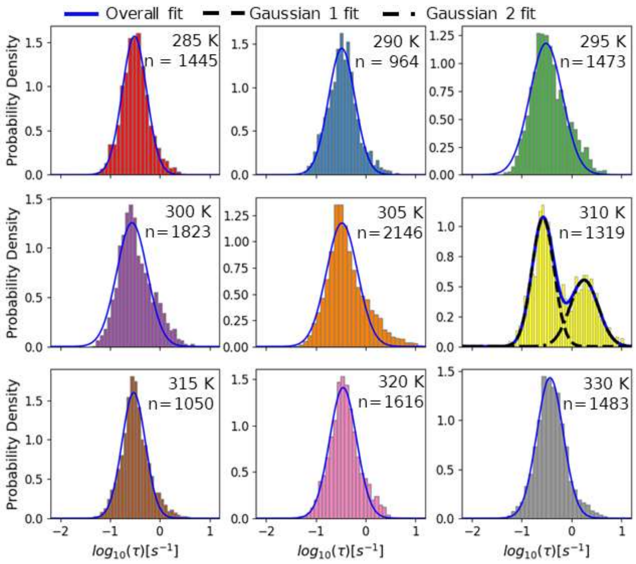

Fig. S14 Histograms of τ value distributions obtained by DXB analysis with 250x4 treatment.

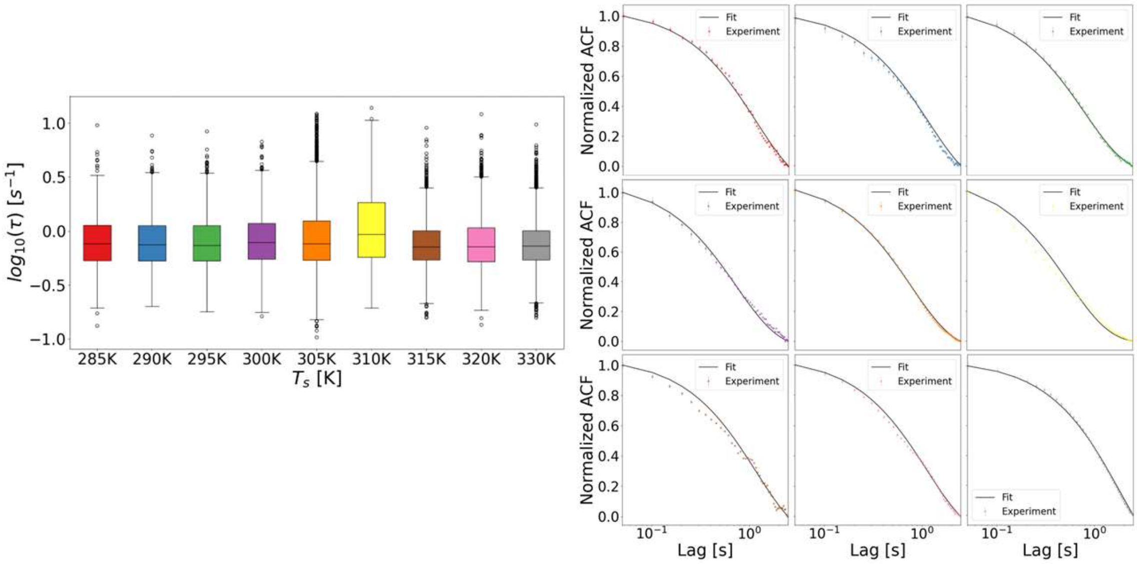

Fig. S15 Boxplots for τ values and average ACF curves of DXB analysis by 100x10 treatment. ACF colors match boxplots: red: 285 K, blue: 290 K, green: 295 K, purple: 300 K, orange: 305 K, yellow: 310 K, brown: 315 K, pink: 320 K, gray: 330K.

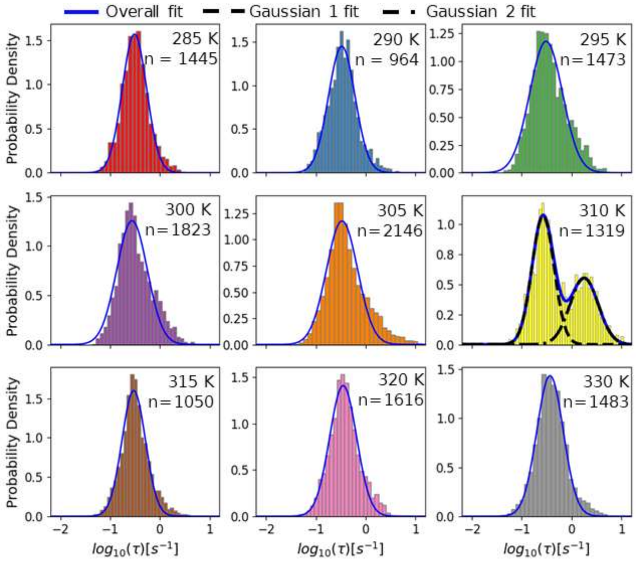

Fig. S16 Histograms of τ value distributions obtained by DXB analysis with 100x10 treatment.

Table S7 Sectioned 250 x 4 results of nonparametric Wilcoxon-Mann-Whitney rank sums tests for the null hypothesis that distributions at different temperatures show statistically significant differences. The shorthand n.s ($p > 0.05$), * ($0.01 < p < 0.05$), ** ($0.001 < p < 0.01$), and *** ($p < 0.001$) is used for clarity.

**T [K]**	**285**	**290**	**295**	**300**	**305**	**310**	**315**	**320**	**330**
**285**	1.0	-	-	-	-	-	-	-	-
**290**	**.	1.0	-	-	-	-	-	-	-
**295**	*	n.s.	1.0	-	-	-	-	-	-
**300**	*	***	***	1.0	-	-	-	-	-
**305**	***	***	***	***	1.0	-	-	-	-
**310**	***	***	***	***	***	1.0	-	-	-
**315**	n.s.	***	***	n.s.	***	***	1.0	-	-
**320**	***	***	***	***	*	***	***	1.0	-
**330**	***	***	***	***	n.s.	***	***	*	1.0

Table S8 Sectioned 100 x 10 results of nonparametric Wilcoxon-Mann-Whitney rank sums tests for the null hypothesis that distributions at different temperatures show statistically significant differences. The shorthand n.s ($p > 0.05$), * ($0.01 < p < 0.05$), ** ($0.001 < p < 0.01$), and *** ($p < 0.001$) is used for clarity.

T [K]	285	290	295	300	305	310	315	320	330
**285**	1.0	-	-	-	-	-	-	-	-
**290**	n.s..	1.0	-	-	-	-	-	-	-
**295**	n.s.	n.s.	1.0	-	-	-	-	-	-
**300**	*	*	*	1.0	-	-	-	-	-
**305**	*	*	***	n.s.	1.0	-	-	-	-
**310**	***	***	***	***	***	1.0	-	-	-
**315**	***	***	***	***	***	***	1.0	-	-
**320**	**	*	n.s.	***	***	***	n.s.	1.0	-
**330**	*	n.s.	n.s.	***	***	***	n.s.	n.s.	1.0

4. Analogous Plot to Figure 7 With G2 Values

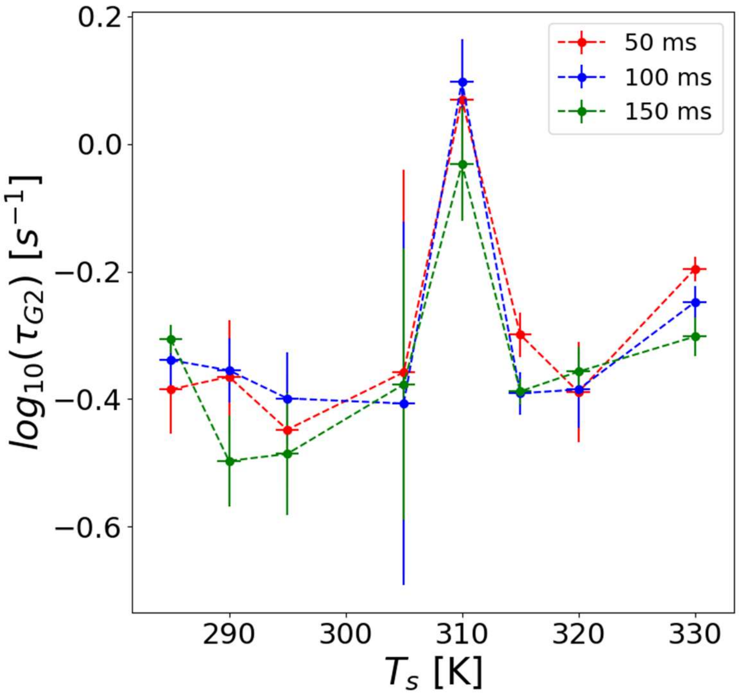

Fig. S17 Expectation values for τ given by the average of G2 components of the histogram fits. Error bars represent variance from the G2 fit along the y-axis and experimentally observed temperature variation (±1) along the x-axis.
